# Drug Repurposing Approach, Potential Drugs, and Novel Drug Targets for COVID-19 Treatment

**DOI:** 10.1155/2021/6631721

**Published:** 2021-04-22

**Authors:** Zemene Demelash Kifle, Akeberegn Gorems Ayele, Engidaw Fentahun Enyew

**Affiliations:** ^1^Department of Pharmacology, School of Pharmacy, College of Medicine and Health Science, University of Gondar, Gondar, Ethiopia; ^2^Department of Pharmacology and Clinical Pharmacy, School of Pharmacy, College of Health Science, Addis Ababa University, Addis Ababa, Ethiopia; ^3^Department of Human Anatomy, School of Medicine, College of Medicine and Health Sciences, Gondar, Ethiopia

## Abstract

Novel coronavirus first appeared in Wuhan, China, in December 2019, and it speedily expanded globally. Some medications which are used to treat other diseases seem to be effective in treating COVID-19 even without explicit support. The existing drugs that are summarized in this review primarily focused on therapeutic agents that possessed activity against other RNA viruses such as MERS-CoV and SARS-CoV. Drug repurposing or repositioning is a promising field in drug discovery that identifies new therapeutic opportunities for existing drugs such as corticosteroids, RNA-dependent RNA polymerase inhibitors, interferons, protease inhibitors, ivermectin, melatonin, teicoplanin, and some others. A search for new drug/drug targets is underway. Thus, blocking coronavirus structural protein, targeting viral enzyme, dipeptidyl peptidase 4, and membrane fusion blocker (angiotensin-converting enzyme 2 and CD147 inhibitor) are major sites based on molecular targets for the management of COVID-19 infection. The possible impact of biologics for the management of COVID19 is promising and includes a wide variety of options such as cytokines, nucleic acid-based therapies targeting virus gene expression, bioengineered and vectored antibodies, and various types of vaccines. This review demonstrates that the available data are not sufficient to suggest any treatment for the eradication of COVID-19 to be used at the clinical level. This article aims to review the roles of existing drugs and drug targets for COVID-19 treatment.

## 1. Introduction

COVID-19 virus belongs to a family of viruses that can cause various symptoms, including fever, breathlessness, pneumonia, and pulmonary infections [1]. On 29 December 2019, the WHO used the term 2019 novel coronavirus or 2019-nCoV to refer to coronavirus in Wuhan, China, that attacks the lower respiratory tract of patients with pneumonia [2]. Later on, February 11, 2020, WHO gave an official name as coronavirus disease 2019 abbreviated as COVID-19. In this name, “CO” stands for “corona,” “VI” for “virus,” and “D” for the disease [3, 4]. As of October 2020, over 39,262,512 cases of COVID-19 have been documented worldwide with nearly 1,104,206 deaths, and 29,423,118 were recovered [2, 5].

Regarding its features, coronavirus is an enveloped ribonucleic acid (RNA) virus, from the genus betacoronavirus, that is distributed in birds, humans, and other mammals [6]. Coronavirus comprises four subfamilies: delta, gamma, beta, and alpha coronaviruses. Among these subfamilies, alpha- and betacoronavirus originate from mammals, specifically from bats; gamma- and delta-viruses originate from pigs and birds [3]. Coronavirus has a single-stranded RNA genome that is enclosed with an enveloped structure and the shape is either spherical or pleomorphic [6].

Different viral agents are linked to an increased risk of more severe disease course and respiratory complications in immune-compromised patients [7]. Even though several investigations have been done on the treatment of COVID-19 in different parts of countries, there is a lack of reviews that comprehensively express novel targets for the management of COVID-19 infection in a different country. Thus, this review summarizes the roles of existing drugs, potential drugs, and novel drug targets for COVID-19 treatment.

## 2. Drug Repurposing for COVID-19

Currently, several vaccines and therapeutic agents are in use for COVID-19 infection prevention and treatment. Vaccines such as Pfizer/BioNTech, Moderna, Johnson & Johnson's Janssen, AstraZeneca are authorized and recommended for COVID-19 infection prevention [8–10].

The whole world is worrying about COVID-19 infection due to the lack of adequate care and public health emergencies. Several governmental and nongovernmental organizations are making exertions to offer COVID-19 patients with quarantine and rapid diagnosis, along with studies to find an appropriate cure that can monitor and avoid the current dangerous effects of the disease [11]. Efforts are made from the day that the virus has erupted which includes drug repurposing and others. Drug repurposing or repositioning is an auspicious field in the development and discovery of therapeutic agents that identifies new therapeutic options for existing drugs [11, 12]. Several established antiviral medications, formerly discovered or used in the management of malaria, MERS, and SARS are being tested for COVID-19 treatment and some of them are being used in clinical trial treatments for COVID-19 infection [13]. This narrative summarizes the current evidence of major potential COVID-19 therapies, repurposed or new drugs, and offers an overview of current clinical practice and recommendations for this new coronavirus pandemic treatment.

### 2.1. RNA-Dependent RNA Polymerase Inhibitors

Among the most fascinating and exciting drug targets for SARS-CoV-2 are the RNA-dependent RNA polymerase (RdRP) and a viral enzyme for viral RNA replication in host cells. Since RdRP is a viral enzyme without host cell homologs, it is possible to produce selective SARS-CoV-2 RdRP inhibitors that have increased potency and fewer off-target effects. Two such nucleoside analogs, remdesivir and favipiravir, are currently being tested in clinical trials for the management of hospitalized COVID-19 infected patients [14].

A recent study has revealed that remdesivir is a potential agent for the treatment of COVID-19 [15]. Remdesivir produces its effect by inhibiting the RNA polymerase and integrating it into nascent viral RNA. This leads to two important outcomes such as termination of the viral RNA chain and subsequently inhibits the viral genome replication. The previous finding revealed that remdesivir was not effective for the treatment of Ebola. However, it provides the safety of remdesivir in humans, which permitted the remdesivir clinical trial in the treatment and protection of COVID-19 without delay [16]. According to an in vivo study in mice, the treatment of MERS-CoV infected mice with remdesivir revealed a reduction in lung viral load, recovery of lung function, and reduce in lung tissue damage [17]. Similarly, both in vivo and in vitro studies showed promising activity of remdesivir in the treatment of SARS-CoV and MERS-CoV [18].

Favipiravir is a prodrug that requires conversion to its active form by phosphoribosylation [19]. Favipiravir became a therapeutic agent that showed a promising effect and is agreed for the management of novel influenza on February 15, 2020, in China. At the very epicenter of the pandemic, favipiravir was initially used for the treatment of SARS-CoV-2 in Wuhan. This medication was then approved for emergency use in Italy as the pandemic spread to Europe and later on to many other countries including Moldova, Ukraine, Japan, Uzbekistan, Kazakhstan, and Russia. The latest commercial launches have also occurred in Egypt, Bangladesh, and Turkey. Favipiravir also obtained approval from the Drugs Controller General of India for mild and moderate COVID-19 infections in India in June 2020 [20]. Case-control studies showed that patients who received favipiravir at a faster rate than those who did not have reduced symptoms of pneumonia and fever [19].

Sofosbuvir and daclatasvir are direct-acting antiviral agents against hepatitis C [21]. They have predicted in silico activity against SARS-CoV-2 [22, 23] and therefore are attractive treatment options. In a recent study, sofosbuvir and daclatasvir demonstrated in vitro activity against Huh-7 and Calu-3 cells infected with SARS-CoV [24]. The study also revealed that daclatasvir is effective against in vitro 6 infected cells; however, sofosbuvir does not. Reports of neurological complications associated with COVID-19 are increasing [25, 26]. And sofosbuvir demonstrated protective activity against SARS-CoV-2 infected brain organoids [27].

### 2.2. Ebselen

Ebselen has been previously found to exhibit potent antiviral activity against many viruses including human immunodeficiency virus type 1 (HIV-1) [28], hepatitis C virus (HCV) [29], influenzas A virus [30], and Zika virus [31].

Ebselen has also shown an effect in rescuing liver injury induced by chemical and microbial stimuli [32]. One of the most common findings observed in severe cases of COVID-19 is liver injury [33]. Therefore, treatment with ebselen might add benefits to this particular aspect of the disease. Moreover, ebselen is also found to be effective in managing focal ischemic injury by decreasing IL-6 [34], which can protect SARS-CoV-2 infected patients with venous thrombosis and vascular injury [35]. Nevertheless, the findings of these previous studies give us hope for the therapeutic potential of ebselen in managing COVID-19. Further investigations are required using randomized clinical control trials before they can be included in any treatment regimen. Ebselen already possesses antiviral activity against several viruses and exhibits potent antiviral activity against SARS-CoV-2 via Mpro inhibition [36], repurposing it for SARS-CoV-2 treatment seems to be a reasonable option [37]. Therefore, ebselen can be considered as a potential therapeutic candidate for COVID-19 patients. However, before including it in the treatment guidelines and widespread use as a potential antiviral drug, further studies should be undertaken to establish its efficacy using experimental and clinical studies [38].

### 2.3. Arbidol

Arbidol is a broad-spectrum inhibitor of parainfluenza virus, influenza A and B virus, and other viruses such as the hepatitis C virus [39]. Arbidol is an orally administered drug at a dose of 200 mg for adults, 3 times/day for no more than 10 days [40]. A recent finding showed that arbidol has the potential to inhibit COVID-19 infections/SARS-CoV-2 infection at 10–30 *μ*M concentration. Arbidol has played a preventive role among health professionals as evidenced by the uninfected rate of health care workers in the arbidol which was significantly higher than that of individuals in the nonarbidol group [41]. Arbidol can also induce interferon and immune cells and therefore has a synergistic therapeutic effect against coronavirus when combined with interferons [42].

### 2.4. Interferons

Interferons are a cytokine family that have antiviral properties. Since it possesses antiviral activity tested in both in vivo and in vitro models, it has been wished for as a possible therapeutic agent for the management of COVID-19 infection. The functions as a paracrine as well as autocrine manners to induce the expression of various interferon-stimulated genes that provide antiviral effects to the host cells [43]. Among interferons, interferon-*α* is known to have broad-spectrum antiviral activity. Following activation, multiple effects can be detected including induction of gene transcription. It also inhibits cellular growth, alters the state of cellular differentiation, interferes with oncogene expression, alters cell surface antigen expression, increases phagocytic activity of macrophages, and augments cytotoxicity of lymphocytes for target cells. Interferon-*α* is used to treat hepatitis, though it is reported to inhibit SARS-CoV replication. In vitro test revealed that interferon-*α* is only partially effective against coronavirus [44].

### 2.5. Protease Inhibitor

Medications that are effective against different RNA viruses are being candidates for the treatment of severe acute respiratory syndrome virus 2. As far as this is concerned, the most familiar medications tried in patients infected with severe acute respiratory syndrome virus 2 are IFN, lopinavir/ritonavir, ribavirin, and some others [45]. The protease enzyme is responsible for the proteolytic processing of polyproteins and is required for the virus to replicate within host cells. Lopinavir along with its booster drug, ritonavir, is known for the management of human immunodeficiency. Because of the effective treatment of MERS, the feasibility of the combination of lopinavir/ritonavir was stated in the most recent guideline for the management of COVID-19 infection. Data from in vitro studies revealed that lopinavir/ritonavir can restrict coronavirus replication [46]. Previous studies revealed that lopinavir and ritonavir are capable of inhibiting the 3CL1 proprotease of COVID-19 viral infection [47, 48]. Moreover, several in vivo and in vitro studies revealed the effectiveness of these antivirals for the treatment of MERS and SARS viruses [49, 50]. Recently, the coformulation of lopinavir/ritonavir was tested in patients infected with COVID-19 infection, though it exhibited a slight advantage for improving the clinical outcome of patients infected with COVID-19 [50, 51].

### 2.6. Ivermectin

Ivermectin exerts broad-spectrum antiviral activity against several animal and human viruses, including both RNA and DNA viruses. The antiviral potential of ivermectin against various viruses is mediated by targeting importin *α*/*β*-mediated nuclear transport of HIV-1 integrase and NS5 polymerase; NS3 helicase; nuclear import of UL42; and nuclear localization signal-mediated nuclear import of Cap. As SARS-CoV-2 is an RNA virus, the antiviral activity of ivermectin may be mediated through the inhibition of importin *α*/*β*-mediated nuclear transport of viral proteins. The clinical efficacy and utility of ivermectin in SARS-CoV-2-infected patients are unpredictable at this stage, as we are dealing with a completely novel virus [52]. Targeting the viral nuclear transport process could be a potential treatment modality for RNA viruses including COVID-19 since the viral nuclear transport process plays a significant role in the embarrassment of the host's antiviral response and enhancing the viral replication cycle [53].

### 2.7. Melatonin

Melatonin is a hormone secreted from the pineal gland in a 24-hour circadian rhythm, regulating the normal sleep/wake cycle. As a supplement, melatonin has both phase-shifting and sleep-promoting properties. In addition to promoting sleep, the physiologic roles of melatonin include regulation of the secretion of growth hormone and gonadotropic hormones. It also possesses antioxidant activity. Recently, melatonin is being a potential agent in the treatment of viral infections. Leukocyte-secreted melatonin exerts a strong immune-modulatory role [54, 55]. Previous studies revealed that an increase in age is a weak prognostic factor in patients with COVID-19 infection. This is because, in advanced age, both physiological functions and immune responses are diminished as a consequence of age in the elderly; they have more chances to develop serious pneumonia secondary to COVID-19 infection [56]. In the previous study, melatonin has shown a key role in attenuating and preventing the cytokine storm, leading to a reduction in mortality and morbidity which is due to COVID-19 infection [57].

### 2.8. Teicoplanin

Teicoplanin is a glycopeptide antibiotic with a spectrum of activity. It is similar to vancomycin but some coagulase-negative staphylococci are less sensitive to teicoplanin than to vancomycin. Recently, teicoplanin showed significant activity against SARS-CoV through in vitro model. Thus, teicoplanin can serve as a potential therapeutic agent for the treatment of COVID-19 [58]. Teicoplanin has been effective against numerous virus types including SARS-CoV, MERS-CoV, flavivirus, HIV, Ebola, Ebola, and influenza virus [59, 60]. Teicoplanin inhibits the virus replication cycle and the release of viral RNA by acting on the early step of the viral life cycle (constraining the low pH cleavage of the viral spike protein). This activity indicates the target sequence that serves as a cleavage site for cathepsin L is conserved among SARS-CoV spike protein [58]. Teicoplanin at a concentration of 1.66 *μ*M can inhibit 50% of the virus (in vitro), which was lesser than the concentration reached in human serum (8.78 *μ*M) [58].

### 2.9. 5-Alpha Reductase Inhibitors

Finasteride and dutasteride are the two main 5-alpha reductase inhibitors used in clinical practice and studied for safety and effective profile in the long run [61–63]. The rationale for their use is based on the blockage of conversion of testosterone into 5alplha-DHT and mitigation of transmembrane protease, serine 2 (TMPRSS2) expression [64–66], eventually hampering the overrepresentation of males, and particularly bald ones, in severe COVID-19. Their benefits may be exhibited if used as a preventive strategy or during the first stage of COVID-19 and have demonstrated correlations with lower severity, although causality could not be established [67, 68]. There is one clinical trial currently testing dutasteride in COVID-19 [69].

### 2.10. Spironolactone

Spironolactone, a safe antihypertensive and antiandrogenic drug used since 1959 that acts as a potassium-sparing diuretic drug by antagonizing mineralocorticoid receptors, tends to disclose favorable patterns of the RAAS and ACE2 expression, reduces TMPRSS2 activity due to its antiandrogenic activity, and may prevent acute lung injuries due to its pleiotropic effects [70, 71].

Among therapeutic targets for SARS-CoV-2, a long-used and safe mineralocorticoid and androgen receptor antagonist, with eﬀective antihypertensive, cardioprotective, nephroprotective, and antiandrogenic properties may oﬀer pleiotropic actions in diﬀerent sites to protect from COVID-19. Current data shows that spironolactone may concurrently mitigate abnormal ACE2 expression, correct the balanced membrane-attached and free circulating ACE2 and between angiotensin II and angiotensin (1–7) (Ang-(1–7)), suppress androgen-mediated TMPRSS2 activity, and inhibit obesity-related RAAS dysfunctions, with consequent decrease of viral priming. Hence, spironolactone may protect SARS-CoV2 and has suﬃcient plausibility to be clinically tested, particularly in the early stages of COVID-19 [70].

### 2.11. Sildenafil/Tadalafil

The PDE5 inhibitor sildenafil citrate is a vasodilator that was approved in 1998 for treating erectile dysfunction and more recently received an indication for pulmonary arterial hypertension and idiopathic pulmonary fibrosis [72]. Sildenafil is currently under investigation in a phase 3 trial in patients with COVID-19 (NCT04304313), which will help clarify its therapeutic potential [73]. The goal of sildenafil treatment is to prevent or perhaps block the progression of fibrosis and to improve respiratory parameters in patients. Tadalafil has also shown PDE5 inhibition with an IC_50_ of 5 nM. It possesses high selectivity for PDE5 over PDE1–4 and PDE6. In particular, tadalafil is more selective against PDE5 than PDE6, whereas sildenafil shows similar potency to inhibit PDE5 and PDE6 [74]. Theoretically, the once-daily use of tadalafil, which is a long-acting drug, could be useful to improve tissue vascularization and to combat fibrosis. Tadalafil is probably less effective in the acute phase but could lend itself to use once daily for possible prevention in patients with erectile dysfunction who are not interested in sexual activity, and for similar purposes, it could be administered to all discharged patients recovering from COVID-19 [75].

### 2.12. Statins

Statins are known for their pleiotropic anti-inflammatory effects, including augmentation of ACE2 expression and inhibition of the Toll-like receptor- (TLR-) MYD88-NF-kB pathway in vitro [76]. In COVID-19 patients, the same anti-inflammatory activity might improve outcomes in those patients with increasingly severe illness, worsening respiratory failure, and increasing D-dimer and IL-6 levels: all factors associated with increased mortality [77, 78]. Earlier studies suggested the possible effectiveness of statin therapy in decreasing influenza-related hospitalizations and deaths. During the 2009 H1N1 pandemic, statin therapy was associated with reduced disease severity among hospitalized patients [79–82]. Because of their pleiotropic actions, statins have been purposed to reduce the occurrence and severity of acute respiratory distress syndrome states and the effects of endotoxin in lung injury [83,84], acting against COVID-19, particularly in the second and third stages [85, 86].

### 2.13. N-Acetylcysteine

N-Acetylcysteine (NAC), a precursor of the antioxidant glutathione, has been used to loosen thick mucus in the lungs and treat acetaminophen overdose for decades. It also boosts the immune system, suppresses viral replication, and reduces inflammation. Despite these valuable features, NAC has been mostly overlooked throughout SARS-Cov and MERS-Cov epidemics, as well as the current COVID-19 pandemic [87].

NAC has been demonstrated to inhibit NF-*κ*B, as well as the replication of human influenza viruses (H5N1, Vietnam/VN1203 strain) in human lung epithelial cells in a dose-dependent manner (5 to 15 mM). It also reduced the production of proinflammatory cytokines (IL-8, CXCL10, CCL5, and IL-6), thus decreasing the chemotactic migration of monocytes [88]. In addition, NAC has also been shown to inhibit replication of other viruses, such as human HIV [89], and respiratory syncytial virus (RSV) [90]. This means that, theoretically, NAC has the potential to inhibit SARS-Cov-2 as well because of its ability to negatively regulate NF-*κ*B [87].

### 2.14. Bromhexine Hydrochloride

Bromhexine hydrochloride is approved in many countries as a commonly used over-the-counter expectorant for both adults and children and has been marketed since 1963 [91, 92]. It is characterized by low side effects and relatively low cost [93]. Considering all these characteristics, bromhexine hydrochloride could be an ideal candidate as a potential COVID-19 treatment [93, 94].

According to the results of cell experiments, the IC_50_ of bromhexine hydrochloride on TMPRSS2 protease is 0.75 *μ*M, and the target cell concentration is about 308.62 ng/mL [95]. The pharmacokinetic data for oral bromhexine hydrochloride show that the adult maximum blood concentration (*C*_max_) with oral administration of 8 mg bromhexine hydrochloride is 22.50 ± 7.50 *μ*g/L [96], and the concentration in the parenchymal tissue of the lung is 54–132.75 ng/mL, far less than the concentration of target cells [91]. Because the new pharmacokinetic parameters of bromhexine hydrochloride are proportional to an oral dose of 8–32 mg [96], the pulmonary concentration with 32 mg of the drug would be 216–531 ng/mL (median 373.5 ng/mL), which could achieve the target cell concentration to inhibit TMPRSS2. In adults, Yong et al. suggested an oral dose of bromhexine hydrochloride up to 96 mg per day in clinical practice [97], to maximize the drug concentration in blood. We did not find reports of pediatric use experiences [98].

### 2.15. Chloroquine Phosphate or Hydroxychloroquine

Chloroquine (CQ), an old antimalarial agent with anti-inflammatory and immunomodulatory activities, has gained significant interest as a potential therapeutic option for the management of COVID-19 associated pneumonia in multicenter clinical trials conducted in China. In early February, Wang et al. demonstrated potent in vitro activity of chloroquine against SARS-CoV-2 with an EC_50_ at 48 hours of 1.13 *μ*M in Vero E6 cells. As an alternative, hydroxychloroquine (HQ), a compound that differs from chloroquine only by a single hydroxyl group, has garnered interest. It is perceived as having better tolerability than chloroquine, which has led to long-term usage in rheumatological disorders [3, 99]. These data were consistent with previous data for chloroquine's inhibitory activity against SARS-CoV-1 and MERS-CoV in various cell lines, where EC_50_ values of 1–8.8 and 3.0 *μ*M were demonstrated, respectively. Chloroquine and hydroxychloroquine have a long-standing history in the prevention and treatment of malaria and the treatment of chronic inflammatory diseases including systemic lupus erythematosus and rheumatoid arthritis [100].

Previous studies reported that CQ/HCQ possess a broad spectrum of antiviral effects on a variety of viruses as diverse as (HIV) Marburg virus, Zika virus, dengue virus, Ebolavirus, and SARS-CoV-1. CQ and HCQ can interfere with the binding of viral particles to their cellular cell surface receptor or the pH-dependent endosome-mediated viral entry of enveloped viruses to inhibit the viral cycle. They can also interfere with the posttranslational modification of viral proteins or impair the proper maturation of viral protein by pH modulation. In addition, CQ and HCQ can regulate the immune system by affecting cell signaling and the production of proinflammatory cytokines [101]. Moreover, CQ on the growth of SARS-CoV-2 in vitro and an early clinical trial conducted in COVID-19 Chinese patients showed a significant effect, in terms of both clinical outcome and viral clearance. Chinese experts recommend that patients diagnosed with mild, moderate, and severe cases of COVID-19 pneumonia and without contraindications to it be treated with 500 mg chloroquine twice per day for ten-day treatment duration. HCQ (an analog of chloroquine) has been demonstrated to have an anti-SARS-CoV activity in vitro. Its clinical safety profile is better than that of CQ (during long-term use) and allows higher daily dose and has fewer concerns about drug-drug interactions. HC/HQ alone and in combination with azithromycin was highly effective in clearing viral nasopharyngeal carriage within six days in COVID-19 subjects [102].

### 2.16. Corticosteroids

Corticosteroids have been used for SARS-CoV-1 and MERS-CoV infections in a few studies. The use of corticosteroids drugs for these viral infections including influenza A had been associated with decreased mortality. But their use now became controversial because of their effect on the suppression of the immune system. Though, patients with COVID-19 infection taking corticosteroids for long-term maintenance dose did not show increment in the incidence of development of severe or critical pneumonia. Meta-analysis studies showed that among corticosteroids, dexamethasone, methylprednisolone, hydrocortisone, and prednisolone were tested for their role in the management of COVID-19 infection [103]. A study conducted in UK showed that dexamethasone has effectively decreased deaths by 1/3 in patients infected with COVID-19 [104]. Similarly, a study conducted on hospitalized patients revealed that the mortality rate of COVID-19 infected hospitalized patients was significantly (*p* < 0.001) varied reliant on respiratory support. Dexamethasone showed a reduction in deaths by 1/3 in patients getting invasive mechanical ventilation (*p* < 0.001, 29.00% vs. 40.70%), by 1/5 in patients getting oxygen without invasive mechanical ventilation (*p*=0.002, 21.50% vs. 25.00%). However, dexamethasone did not decrease mortality in patients not getting respiratory support (*p*=0.14, 17.00% vs. 13.20%) [105]. The mechanism behind corticosteroids in COVID-19 remains to be an objective of ongoing clinical trials [106]. However, their anti-inflammatory effect can decrease systemic inflammation, reduce fluid accumulation in the lung tissue, and prevent more alveolar damage from spreading, thus improving hypoxia and decreasing the risk of respiratory failure [107].

## 3. Targets for New Drug

Since the available drugs are not much successful for COVID-19, a search for new drug/drug targets is underway. Therefore, the following are major sites based on molecular targets [108].

### 3.1. Blocking Coronavirus Structural Protein

The SARS-CoV-2 (SARS-CoV-2S) surface spike glycoprotein plays a significant role in viral infection, beginning with the identification of the host cell surface receptor and binding the viral envelope to the host cells for fusion and infiltration. Spike glycoprotein includes host receptors for ACE2 for entry into host cells, where spike receptor recognition and attachment are recognized and attached [109]. F26G19, CR3014, S230, CR3022, and F26G18 are recently developed therapeutic antibodies for the management of COVID-19 infection that target spike-protein [110].

### 3.2. Targeting Viral Enzyme

The main protease, 3C-like protease (3CL (pro)), and papain-like protease (PLpro) are drug targets among coronaviruses [111]. By identifying the P1 and P1–P4 sites, 3CLpro cleaves 11 sites in the polyproteins, with the recognition sequence Leu-Gln (Ser, Ala, Gly), including its N- and C-terminal autoprocessing sites. A recent study has shown that through the subsite cooperation of Phe P2 and Phe P3 P3, 3CLpro cleaves its C-terminal autoprocessing site. On the other hand, PLpro is involved as an evasion mechanism against host antiviral immune responses in cleaving posttranslational protein modifications on host proteins [49]. Therefore, in the processing of polyproteins and viral replication, both PLpro and 3CL (pro) play important roles and they are a potential target. Therapeutic agents such as cyclohexyl methyl (IC_50_ = 0.71 *μ*M), *α*-Ketoamide inhibitors (IC_50_ = 11.4 ± 1.4 *μ*M), pyrazolone derivatives (IC_50_=5.8 ± 1.5 *μ*M), and 1,3,4-oxadiazole disulfide (0.516 ± 0.06 *μ*M) have shown a significant in vitro SARS-CoV-2 inhibitory activities by targeting 3CL(pro) protease. [49].

Unlike that of 3CL (pro)), targeting the PLpro is found to be challenging for pharmaceutical companies that scramble to discover a drug against COVID-19. The encounter behind this is that PLpro recognizes the C-terminal sequence of ubiquitin of both the virus and host which in turn inhibits host-cell deubiquitinases and lacks selectivity [112].

### 3.3. Dipeptidyl Peptidase 4 as a Target

Dipeptidyl-peptidase 4 (DPP4) is a glycoprotein of 110 kDa and is expressed on the surface of a variety of cells. DPP4 has a role as a peptidase, cutting N-terminal dipeptides, leading to degradation of several compounds such as dipeptides from a variety of substrates, including neuropeptides, growth factors, incretin hormones, and cytokines [113]. DPP-4 is expressed on cells inside such as interstitial (endothelial cells), type I and II cells (lung parenchyma), mononuclear lymphoid cells, and macrophages cells. Moreover, increased DPP4 immunostaining in alveolar macrophages, alveolar epithelial, and type I and II alveolar cells was reported in patients with chronic lung disease. This suggests that overexpression of DPP4 on vascular endothelia and immune cells could attribute to CoV-linked disease [114].

Studies suggested that the S1 domain of SARS-CoV-2 S protein aids as the viral binding site and interrelates with DPP4. The interaction of DPP4 with MERS-CoV S protein triggers signals that hinder the activation of macrophages. Macrophage infection secondary to MERS-CoV S protein pseudotyped particles also defeats the macrophage replies through elevating the synthesis of LPS-induced immunosuppressive cytokine IL-10 and through dropping their capability of synthesizing IL-6 and TNF alpha in naive and LPS-activated THP-1 macrophages. Above all inhibition of DPP4 through sitagliptin/siRNA, a DPP4 inhibitor reduced the activity of the MERS-CoV S protein on IL-1 receptor-associated kinase, peroxisome-proliferator-activated receptors, and IL-10 suggesting that hindering the DPP4 receptors could have a significant role in the modulation of the COVID-19 infection immune response [115].

### 3.4. Membrane Fusion Blocker (Angiotensin-Converting Enzyme 2) is a Target for COVID-19 Treatment

The first stage of viral infection is the identification of receptors by the S1 subunit of the coronavirus spike protein, followed by receptor-binding domain binding to the receptors. Angiotensin-converting enzyme 2 (ACE2) is used by SARS-CoV-2 and SARS-CoV as a receptor to mediate viral entry into the target cell. The early stages of in vitro SARS-CoV-2 infection can be suppressed by human recombinant soluble ACE2, which has been studied in clinical trials for the management of acute pulmonary injury and pulmonary hypertension, indicating that therapeutic agents acting on the ACE2 pathway may have a beneficial role in the management of COVID-19 infection [116].

In vivo studies have shown that chronic treatment with angiotensin II type 1 receptor antagonists such as olmesartan, losartan, and lisinopril facilitates renal and cardiac angiotensin-converting enzyme 2 receptors gene expression [117]. However, following the entry of severe acute respiratory syndrome virus into respiratory epithelial cells inhibits the effect of angiotensin-converting enzyme 2 receptors thereby increasing the levels of angiotensin II. This leads to severe lung impairment [118]. Though, treatment with angiotensin receptor blocker and angiotensin-converting enzyme inhibitors may be crucial for the survival of a patient infected with COVID-19 through attenuating the cardiac stress and reduce vasoconstriction and profibrotic activity of angiotensin 2 in alveolar capillaries [119].

### 3.5. Membrane Fusion Blocker (CD147) as a Target for COVID-19 Treatment

In addition to ACE2, CD147 plays a significant role in the pathogenesis of SARS-COV-2 to invade host cells. Therefore, therapeutic agents that affect the interaction of the CD147 expression and spike protein, including progenitor/stem cells, can inhibit the viral dissemination and invasion amongst other cells. The beneficial effect of azithromycin in the management of patients with COVID-19 infection is linked with its effect with interfering with the ligand of CD147. Moreover, besides the potential invasion outcome, treatment with azithromycin reduces the expression of certain metalloproteinases (downstream to CD147) and induces antiviral responses in rhinovirus-infected primary human bronchial epithelial infections, minimizing viral replication and releasing viral infection [120].

## 4. Nonpharmacologic Treatment and Traditional Herbal Medicines for COVID-19

Recently, debatable thoughts between the COVID-19 infection and immune system are forwarded. Different studies recommended that reducing stress, having regular exercise, eating a balanced diet with fruits and vegetables, avoiding smoking, and getting enough sleep are some of the suggested approaches to trigger the immune system [121].

The use of traditional medicines for the management of the infectious disease has increased because of several reasons including the participation of different concerned bodies in the production and investigations of herbal-based medicines [122, 123]. Recently, several studies were conducted in elucidating the medicinal values of herbal medicines for the management of COVID-19 [124]. Previously, plant-derived herbal medicines have been employed for the management of different outbreaks including H1N1 influenza and SARS [125, 126]. Currently, several countries such as South Korea and China have agreed on guidelines on the use of traditional medicine for the management of COVID-19 [127]. In China, eighty-five percent of COVID-19 infected patients received traditional medicine [124]. A recent study revealed that traditional medicines are supposed to competitively target angiotensin-converting enzyme 2 receptor similar to SARS-CoV- 2 and SARS-CoV. This revealed the possibilities of traditional medicine for the management of SARS-CoV-2 [128]. Plant-based traditional medicines such as *Radix platycodonis, Agastache rugosa, Saposhnikoviae divaricata, Lonicerae japonicae flos, Astragalus membranaceus, Rhizoma Atractylodis Macrocephalae, Glycyrrhiza uralensis, Atractylodis Rhizoma, Fructus forsythia,* and *Cyrtomium fortunei J. Sm* were the most commonly used herbal medicine for the management of COVID-19 in China [129].

## 5. Conclusion

This literature review and analysis were conducted based on newly published studies on the treatment and protection of COVID-19 infection. This review demonstrates that the available data are not sufficient to suggest any treatment for the eradication of COVID-19 to be used at the clinical level. The drug-repurposing trial summarized in the present review mainly concentrated on agents presently identified to be effective against RNA viruses such as influenza, SARS-CoV, Ebola, MERS-CoV, and HCV. The possible impact of biologics for the management of COVID-19 infection is auspicious and includes a wide variety of options such as cytokines, nucleic acid-based therapies targeting virus gene expression, bioengineered and vectored antibodies, and different types of vaccines. Thus, blocking coronavirus structural protein, targeting viral enzyme, dipeptidyl peptidase 4, and membrane fusion blocker (angiotensin-converting enzyme 2 and CD147 inhibitor) are major sites based on molecular targets for the management of COVID-19 infection. The evidence summarized in this review provides a strong intellectual groundwork for the development and discovery of vaccines and drugs against COVID-19 infection.

## 6. Limitations of the Review

Even if this review has its strengths, such as the inclusion of many recent published research works and critically appraising the selected studies, it is not without limitations. The limitations of the current review are the complete reliance on previously published researches.

## Abbreviation

2019 nCOV: 2019 novel coronavirus
ACE-2: Angiotensin-converting enzyme 2
COV: Coronavirus
CQ: Chloroquine
DPP4: Dipeptidyl-peptidase
HCQ: Hydroxychloroquine
HIV: Human immunodeficiency virus
MERS-COV: Middle east respiratory syndrome coronavirus
NAC: N-Acetylcysteine
RdRP: RNA-dependent RNA polymerase
RNA: Ribonucleic acid
RBD: Receptor binding domain
TMPRSS2: Transmembrane protease, serine 2.
